# The Effects of Dentition on Nursing Children

**Published:** 1856-09

**Authors:** M. Trousseau


### *The Effects of Dentition on Nursing Children.* By M. Trousseau.

[Clinical Lecture, delivered at the Hotel Dieu. Translated for the Boston Medical and Surgical Journal. From the Gazette des Hopitaux, Dec., 1855.]

The most elementary questions in medicine are often the least understood. It would seem, at first sight, that we need not much concern ourselves about the trifles which daily swarm beneath the feet of the practitoner; but remember that Stoll has written a chapter entitled *De quibusdam magni momenti minutiis*, and learn early to neglect nothing.

The infant has twenty teeth, the adolescent twenty-eight, the adult thirty-two. The evolution of the twenty teeth of the infant is not completed before the thirtieth to thirty-sixth month; but they are only tempor ry, for, at the age of seven years, he begins to lose them, exchanging them for others which are more durable. This process is normally accomplished at thirteen or fourteen years. Except the great king, who formed an exception to everything, and who was born, it is said, with two teeth, the infant comes into the world with defenceless jaws, and it is not till towards the eighth month that the first milk teeth appear. But since the laws of nature are capricious, it often happens that one infant has teeth at four months, while another has none at the end of a year; hence no limits can be fixed. Generally, the two middle incisors of the lower jaw first appear, and I anticipate a stormy dentition whenever I see a child begin that process by the upper teeth. These two first teeth appear together, with an interval of twenty-four hours, forty-eight hours, four days, and sometimes a week, between them, but always *together*, remember, and they are the only ones which present themselves in this manner. Six weeks or two months afterwards, the two superior middle incisors make their appearance, not together, but at the distance of eight, fifteen or thirty days from each other. The process of dentition is thus very rapid for the first two teeth and more slow for the others.

Meanwhile, two other teeth are about to protrude—the lateral incisors of the upper jaw—very soon, one or two months, after the upper middle incisors. Towards the end of one year, the child has six teeth, and whereas he began with two lower, he has finished with four upper.

The teeth of children appear in *groups; dentes in infantibus catervatim erumpunt:* first group, two inferior, middle incisors, at about eight months; second group, two superior middle incisors, towards ten months; third group, two superior lateral incisors, at one year, more or less; fourth group, two inferior lateral incisors and the first four molars (six teeth in this group, from fourteen to eighteen months); fifth group, four canines, from eighteen to twenty-four months; sixth group, four second and last molars, from thirty to thirty-six months.

The canine teeth appear after the infant has twelve teeth, and when he is from eighteen to twenty-four months old; their evolution lasts from two to three months, sometimes of ten months, then takes places, and at the age of three years, when those of the last group have pierced the gums (the four second molars), the process of dentition is finished.

It is not without object that I have spoken of groups; you will see that a knowledge of this arrangement is very important in respect to weaning. It is a fact worthy of consideration, that immediately after a group of teeth has appeared, there is an interval of rest for the child. Profit, then, by this interval to wean, for the moment is propitious. Do you know what is commonly done? Children are weaned indifferently when they have two, seven, nine, eleven, fourteen teeth; no attention is paid to the number Now, I entreat you to pay close attention to this, otherwise you will lose your little patients by that terrible affection of the intestines, *cholera infantum.*

You will often be consulted as to the time for weaning; never give an opinion, therefore, until after a scrupulous examination of the state of the dentition, and do not authorize the mother to wean her infant until it has six, twelve, or sixteen teeth. Good practitioners will never permit a child to be weaned after the evolution of the first two teeth; the patient is to young; he is ordinarily but eight months old. It is only by careful management that you will succeed after the eruption of the third group; still, if you are strongly urged by the parents, consent, for you have before you a month or six weeks of respite before the evolution of the fourth group. Allow it, then, in case of necessity, but never forget that the child has only six teeth, that he is only a year old, and that artificial alimentation will not always be successful.

The most favorable period for weaning is, beyond all doubt, the interval separating the fourth from the fifth group. The child, in fact, is armed with twelve teeth, eight incisors and four molars, and he has before him a tolerably long time of rest, about two months, during which there is no reason to dread any intestinal trouble, and when the canines begin to appear (which group causes the greatest danger in its evolution), he is accustomed to his new diet, and prepared for the crisis, which he is about to undergo.

Learn, then, to wait until after the fourth group, before weaning. If the health of the mother or nurse, or the circumstances of the family, oblige you to authorize an early weaning, always see that there are six teeth; but if, on the contrary, you are not obliged to yield to considerations of this nature, do not allow weaning until you can count twelve.

Do not imagine that things always go on so regularly. You will see children who have the molars before the incisors, or the superior incisors before the inferior incisors; for although dentition ordinarily takes place in the way I have described, it is no less true that it frequently presents irregularities which greatly perplex the physician who is earnestly watching for an interval of repose. In such a case, do the best which the circumstances will admit of; examine the state of the gums, and have the child weaned immediately after the complete evolution of a tooth, which will prbably be followed by a period of repose, during which you will have leisure to guard against evil consequences.

Among the affections which are common to dentition, the most important, the most grave and the most obstinate are seated in the alimentary canal. A few days before it begins, the infant is restless, wakeful, cries violently, sucks its fingers, bites the nipple, refuses to feed, if it takes supplementary nourishment, and sometimes will not nurse. Its gums are red, and there is a very evident prominence at the points which the teeth are about to pierce; there is cough, the voice is changed, the mucous membrane of the mouth is irritated. From the moment the child has two teeth, the neighboring gums become inflamed, and the protruded teeth will be surrounded by a ring of red and swollen gum.

If you give mercury to a person who has no natural teeth, but who wears an artificial set, you will not see salivation, nor mercurial stomatitis, follow. But if the patient have a single tooth remaining which has escaped destruction, the effects of the mercury are manifested around it. The gum surrounding the tooth will inflame, while the rest of the mouth will be free from disease. The same is true with regard to the first two teeth; their eruption causes no affection of the gums, which, however, swell and become red with the evolution of the second and succeeding groups.

In almost all children the process of dentition is accompanied with diarrhœa. This is sometimes moderate, consisting of three or four dejections only, daily, but it is frequently excessive, with green stools, resembling chopped herbs, or grains of curdled milk, with glairy and sometimes bloody matter. In certain cases marked tenesmus manifests itself, with prolapsus of the rectum. These symptoms, which precede, by several days, the eruption of the tooth, often continue, and even last until the entire group penetrates the gums. If the diarrhœa does not cease, you are aware what treatment should be adopted, and what attention should be paid to the diet. You will restrain and mitigate it as much as possible.

Would you advise weaning during this diarrhœa? No, unless the nurse's milk seems to keep up the intestinal flux.

During the summer season, the injurious effects of dentition are chiefly directed towards the intestines, very rarely upon the air passages. Intestinal derangements, fever, peripneumonic catarrh, and other morbid pulmonary manifestations, occur in the winter.

I must warn you against a popular prejudice which I advise you to oppose on every occasion that offers. You will hear it said again and again that diarrhœa is beneficial to children; believe it not, for too often it will cause the death of your little patient Diarrhœa prepares the way for chronic enteritis, and chronic enteritis debilitates and destroys its victims. On the contrary, restrain the itestinal flux, and you will [illegible] that the other symptoms are much better borne.

In the same way, it is considered highly advantageous to leave untouched the filth which covers the head of a new-born infant. This ridiculous prejudice no longer exists in England or America; let us do away with it here.

When, during dentition, the evacuations are merely more loose than common, without amounting to diarrhœa, this slight derivative effort requires no interference, but it should not be allowed to continue too long.

It has been said that convulsions are common with infants whose bowels are constipated, but do not attack those who have diarrhœa. This is not true. Convulsions almost always accompany diarrhœa, and are prevented by a good state of the bowels.

I call your attention particularly to the diet, as a point of the greatest importance. If you neglect caution in this respect, you will have diarrhœa, followed by enteritis, serious indigestion and eclampsia. Nothing is more common than severe cases of indigestion, aggravated by enteritis, and leading to convulsions; and nothing is more alarming to the parents, who generally lose their senses, and while the domestics or the neighbors run to bring the doctor, the mother, following the advice of some officious gossip, pours hot water over the hands and feet of her infant; he is scalded, and dies from the effects of it. This reminds me of what occurred to an eminent brother-physician, Professor Marjolin, during the course of a typhoid fever, which threw him into a state of profound stupor. They applied to his legs napkins wet with water at a temperature of 158° Fahr. Large eschars followed, which were not completely healed for several months.

If convulsions occur, the less you do, the better. The attack, indeed, is most frequently over when you arrive, and although there may be a slight recurrence once or twice during the day, the remembrance of it, only, is left, the day after. If there have been indigestion, administer a laxative, in order to expel any undigested food; allow the child to nurse but little, give it water with some albuminous substance in solution, and in an urgent case, a bath, and you will soon see the alarming train of symptoms disappear. Almost any treatment succeeds in the majority of cases, even the infinitesimal doses of that absurd system—homœopathy.

